# Cardiac herniation presenting as superior vena cava obstruction syndrome after intrapericardial pnemonectomy for locally advanced lung cancer---case report

**DOI:** 10.1186/s13019-021-01439-5

**Published:** 2021-03-31

**Authors:** Gengxu He, Tong Yao, Lei Zhao, Hong Geng, Qiang Ji, Kun Zuo, Yuanzhi Luo, Kai Zhou

**Affiliations:** 1 Department of Thoracic and Cardiovascular Surgery, The First Affiliated Hospital of Hebei North University, Zhangjiakou City, Hebei Province People’s Republic of China; 2 Department of the ECG, The First Affiliated Hospital of the Hebei North University, Zhangjiakou, China

**Keywords:** Cardiac herniation, Pneumonectomy, Superior vena cava obstruction

## Abstract

**Introduction:**

Cardiac herniation is a rare complication after pulmonary surgery, and there are only a few reports about it. We now report a case of cardiac herniation presenting as superior vena cava obstruction after pneumonectomy.

**Case presentation:**

A-52-years old woman diagnosed right pulmonary squamous cell carcinoma was carried out right pneumonectomy, the pulmonary artery and right superior pulmonary vein were dissected and ligated intrapericardial. The patient developed tachycardia arrhythmias, hypotension, followed by loss of consciousness at about 18 h after operation. After resuscitation, the patient was conscious but developed cyanosis of the superior vena cava drainage area, uropenia, and hypotension (80/30 mmHg). Bedside-echocardiography showed that the SVC was obstructed due to thrombus formation. Chest radiography a shift of the heart into right hemithorax. Rethoracotomy was performed and the herniated heart was replaced into the pericardium, and the pericardium was repaired with Gore Tex patch. The patient recovered smoothly after the second surgery.

**Conclusion:**

Cardiac herniation is a rare and fatally complication after thoracic surgery, and the prompt recognition with timely intervention is life-saving. Cardiac herniation is a rare but fatal complication of pneumonectomy. The increasing frequency of surgical resection for locally advanced thoracic carcinoma has led to a renewed emphasis regarding early diagnosis and treatment for cardiac herniation. Here we discuss a case of cardiac herniation presented with acute superior vena cava obstruction syndrome and hemodynamic instability after intrapericradial right pneumonectomy.

## Case report

**Fig. 1:**
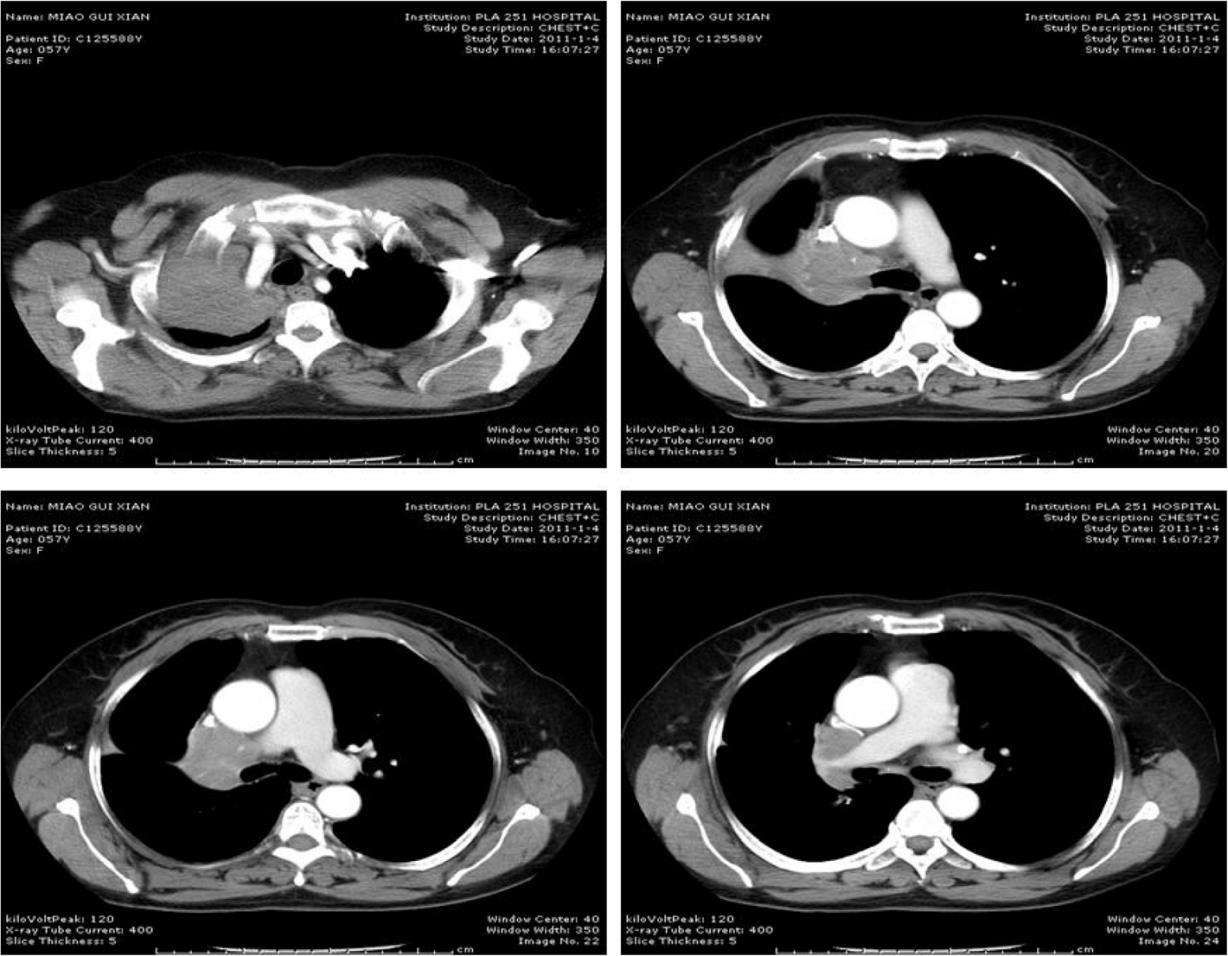
Computed tomography (CT) scan of the chest show atelectasis of right upper lobe tumor invasion into the right main pulmonary artery

**Fig. 2:**
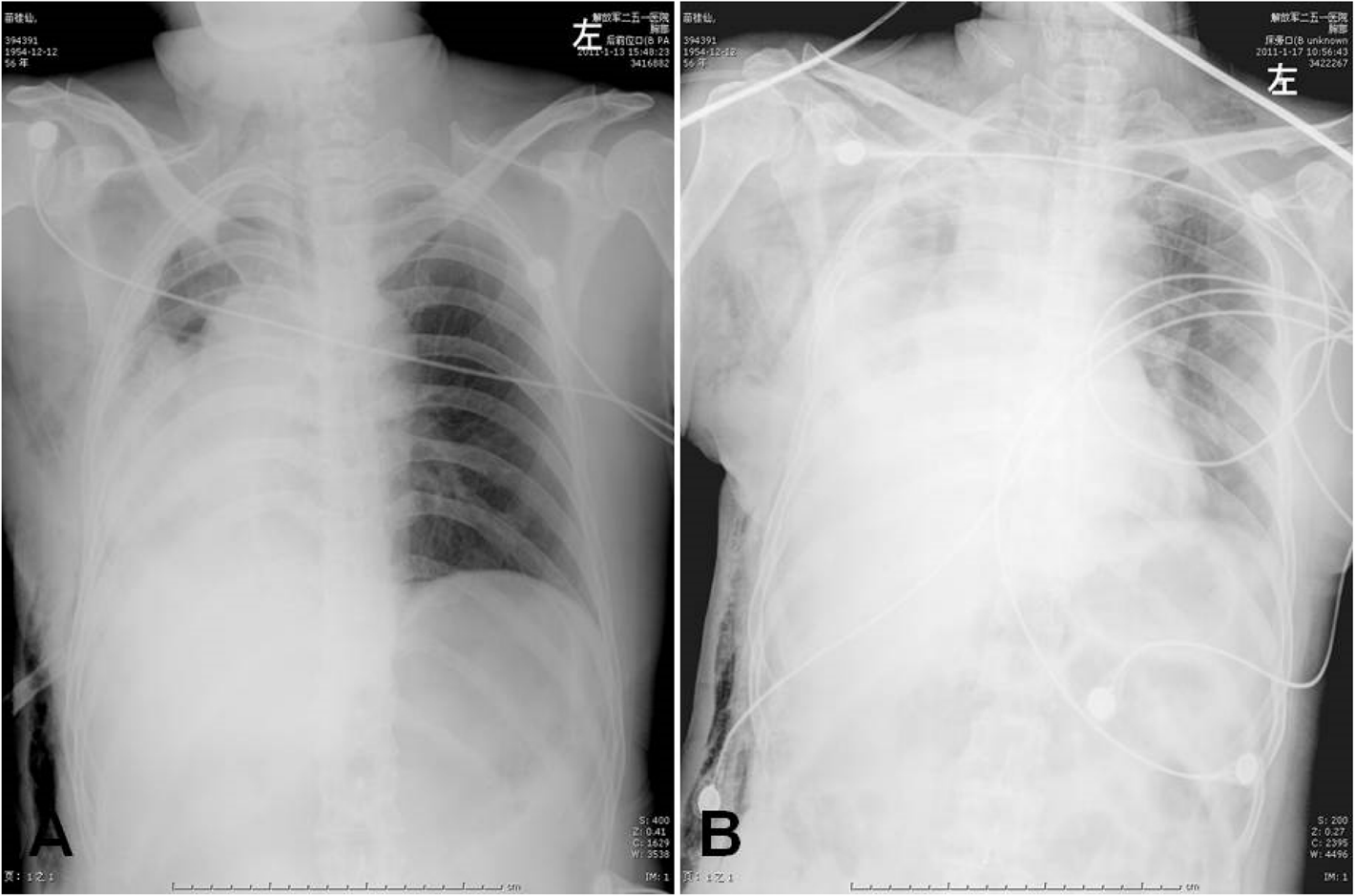
Chest radiography showed a shift of the heart into right hemithorax after 18 h of intrapericardial pneumonectomy (**a**), and the apex of heart returned into normal position after rethoracotomy (**b**)

A 52-year-old woman with a 3 month’s history of cough and hemoptysis consulted a nearby clinic. Chest X-ray showed atelectasis of the right upper lobe caused by locally advanced lung cancer with mediastinal invasion. Bronchoscopic biopsy showed squamous cell carcinoma. Chest computed tomorgraphy (CT) showed atelectasis of right upper lobe tumor invasion into the right main pulmonary artery. The SVC was compressed and narrowed but remained patent (Fig. 1). No significantly enlarged mediastinal lymph nodes were seen. Bronchoscopy revealed an exposed tumor in the right upper bronchus which completely obstructed the lumen. Brain and abdominal CT, and bone scintigraphy revealed no signs of distant metastases. On the basis of these findings, the tumor was evaluated to be resectable by intrapericardial pneumonectomy. The surgery was performed through a right thoractomy. The pericardial was opened from SVC to the level of right upper pulmonary vein 2 cm posterior and parallel to the phrenic nerve. The SVC was isolated from the atrium to the brachiocephalic trunk, which involved SVC denudation at the point of its connection with pericardium. Then the right pulmonary artery was dissected and ligated between the ascending aorta and the SVC. The right upper pulmonary vein was ligated intrapericardially. The right lower pulmonary vein was dissected and ligated outside of the pericardium. Finally the right main bronchus was manipulated using a stapler to complete the right pneumonectomy. No direct tumor invasion into the SVC was detected. The lower part of pericardium was closed interruptedly and the upper defect was left open. The chest was drained using an intercostals tube that was kept clamped and released for half an hour the next morning. The patient was transferred postoperatively to Intensive Care Unit, and the tracheal tube was withdrawn 2 h after operation. About 18 h after operation, the patient was transferred to an ordinary room. Immediately after a short period of hard coughing, the patient developed tachycardia arrhythmias, hypotension, followed by loss of consciousness. After resuscitation, the patient was conscious but developed cyanosis of the SVC drainage area, uropenia, and hypotension (80/30 mmHg). She felt more comfortable in the right decubitus position. Emergency bedside-echocardiography showed that the SVC was obstructed due to thrombus formation. Cardiac herniation into the right pneumonectomy space was detected on repeated chest radiography which showed a shift of the heart into right hemithorax (Fig. 2). Rethoracotomy was performed on the second day after the intrapericardial pneumonectomy. At operation it was found that the heart had rotated into the right hemithorax along the axis of the vena cava, and the SVC was strangulated. The heart was placed back into its normal position. The pericardium was torn from its initial incision to cardiac apex for about 8 cm and this was repaired using a Gore Tex patch. About 5 min after the heart was returned to its normal position, the syndrome of SVC disappeared. The postoperative course was uneventful. The patient has been followed up as tumor-free for more than 3 years.

## Discussion and conclusion

With the increased incidence of primary lung cancer, radical surgery (intrapericaridal pneumonectomy or pneumonectomy combined with partial pericardiectomy) is now being performed more frequently and reports of patients developing cardiac herniation following pneumonectomy have increased.

Several factors lead to postpneumonectomy cardiac herniation. Specifically mechanical ventilation, gastric distention, suction applied to the chest tube or aspiration of the hemithorax, coughing, hyperinflation of the remaining lung, and changes in patients position all may facilitate the dislocation of the heart through the pericardial defect [1]. In our case, the absence of three right points of fixation (SVC and right pulmonary veins at the pericardial junction) presumably enabled the heart to slip through the window and to rotate its tip to the right, and the suddenly increase in abdominal pressure or right hemithorax negative pressure during coughing might also have participated in the rotation. Nowadays, more and more patients with locally advanced lung cancer can be surgically treated with intrapericardial pneumonectomy or local organ resection which may compromise the normal fixation of the heart. Although the pericardial incision which had been closed during operation could be possibly torn again during coughing, and led to cardiac herniation [2]. In our case, the sutured pericardium reopened and torn to the cardiac apex from its initial incision resulted in rotation of the heart. Fortunately the wide open of pericardium was big enough to avoid irreversible hemodynamic dysfunction and only the SVC was obstructed. The incidence of heart displacement has been shown to be independent of the size of the defect and is without side predilection [3]. In order to prevent the dislocation of the heart, the pericardial defect created during intrapericardial pneumonectomy should be repaired with a patch irrespective of the size [4]. As the best material for closure is disputed, Fascia lata is strong; however it requires the creation of an additional wound. Teflon is also strong, but its polyporous, fibrous nature can lead to constrictive pericarditis and infection. Pleural flaps are easy to harvest and provide the simplest and most reliable closure. Polytetrafluoroethylene (PTFE) patches are preferred because of their strength, simplicity, and the low risk of infection. Prolene mesh is strong and porous and its construction may prevent tamponade by allowing pericardial fluid to leak from the restrictive covering [5]. We also recommended that direct suturing should generally be avoided because increased tissue tension and the sutured pericardium could be torn again.

A majority of cardiac herniation occurs within 24 h of surgery and 75% of the events occur intraoperativley [6]. Although there is a report of cardiac herniation occurring 6 months after right pneumonectomy, late herniation occurring 24 h after surgery is rare because of rapid formation of adhesions between the heart and the pericardium [7]. In our case, cardiac herniation occurred about 18 h after surgery. Considering the fact that cardiac herniation is a life-threatening condition with mortality of 50% in recognized cases and 100% in undiagnosed cases [3], early diagnoses is pivotal. The symptoms of cardiac herniation are related to location of pericardial defect created by cardiac dislocation. On the right side, obstructive shock includes kinking or torsion of both SVC and the inferior vena cava with subsequent reduction of cardiac filling causing a decrease in systemic blood pressure, a dramatic increase of central venous pressure and a sudden onset of tachycardia [8]. On the left side, cardiac herniation produces dysrhythmia and acute myocardial ischemia, which can compress or strangulate the ventricular wall due to the pericardial edge leading to hypotension, ventricular fibrillation and infarction [9]. With right cardiac herniation, the characteristic radiologic findings on chest radiograph include: displacement of the heart from the midline with cardiac apex located on the right, an abnormal cardiac contour with a globular right cardiac border protruding into the right chest [10–12], a notch on the side of cardiac vascular pedicle, a clockwise rotation of the Swan Ganz catheter or a kink in the central line at the level of brachiocephalic venous junction [13], and a change in the position of the chest tube. With left cardiac herniation, the classic radiologic findings include: a hemispherical shape of the left heart border, an incisura between the great vessels and the herniated left cardiac chambers, a change in the position of the chest tube, and an empty pericardial sac. Transthoracic echocardiography could also provide an accurate diagnosis [14]. However, in this case, the transthoracic echocardiography did not provide much information other than a SVC obstruction.

In conclusion, prompt recognition with timely intervention is life-saving from cardiac herniation after intrapericardial pnemonectomy. The radiologic finding plays a critical role in the diagnosis of this complication. Patch closure of the cardiac defect during the operation irrespective of the size is necessary to prevent cardiac herniation.

## Data Availability

The datasets used and/or analysed during the current study are available from the corresponding author on reasonable request.

## Abbreviations

SVC: Superior vena cava
CT: Computed tomorgraphy
